# Bridging the gap between self-directed learning of nurse educators and effective student support

**DOI:** 10.4102/curationis.v38i2.1503

**Published:** 2015-11-26

**Authors:** Gisela H. Van Rensburg, Yvonne Botma

**Affiliations:** 1 Department of Health Studies, University of South Africa, South Africa; 2 School of Nursing, University of the Free State, South Africa

## Abstract

**Background:**

Self-directed learning requires the ability to identify one’s own learning needs, develop and implement a plan to gain knowledge and to monitor one’s own progress. A life-long learning approach cannot be forced, since it is in essence an internally driven process. Nurse educators can, however, act as role models to empower their students to become independent learners by modelling their own self-directed learning and applying a number of techniques in supporting their students in becoming ready for self-directed learning.

**Objectives:**

The aim of the article is to describe the manifestations and implications of the gap between self-directed learning readiness of nurse educators and educational trends in supporting students.

**Method:**

An instrumental case study design was used to gain insight into the manifestations and implications of self-directed learning of nurse educators. Based on the authentic foci of various critical incidents and literature, data were collected and constructed into a fictitious case. The authors then deductively analysed the case by using the literature on self-directed learning readiness as departure point. Four constructs of self-directed learning were identified, namely internal motivation, planning and implementation, self-monitoring and interpersonal communication. Supportive strategies were identified from the available literature.

**Results:**

Nine responses by nurse educators based on the fictitious case were analysed. Analysis showed that readiness for self-directed learning in terms of the identified constructs was interrelated and not mutually exclusive of one other.

**Conclusion:**

The success of lifelong learning is the ability to engage in self-directed learning which requires openness to learning opportunities, good self-concept, taking initiative and illustrating independence in learning. Conscientiousness, an informed acceptance of a responsibility for one’s own learning and creativity, is vital to one’s future orientation towards goal-directed learning. Knowledge and understanding of one’s own and students’ self-directed learning abilities are critical for nurse educators. In the nursing profession, it has been shown that self-directed learning by the nurse educators has a direct relationship towards the development of a lifelong learning approach by their students. Supporting students towards becoming self-directed learners throughout their professional life, in turn, will impact directly on the quality of nursing and midwifery practice.

## Introduction

‘The illiterate of the 21st century will not be those who cannot read and write, but those who cannot learn, unlearn, and relearn.’ (Anonymous)

Individuals, families, communities, organisations and countries depend on the ability of people to learn, unlearn and relearn in order to remain relevant and maintain and improve economic growth, democracy, social cohesion, mass media and social integration (Guglielmino & Toffler 2013:4; Walters, Yang & Roslander 2012:9). Hence, individuals need to develop and demonstrate the ability to interpret current and future trends related to their living and working environment to be able to identify their own learning needs. This will enable them to develop and implement a goal-directed plan to gain new knowledge and to monitor their own progress. The drive to learn and change must come from the individual (Chen, Hsu & Hsieh 2012:143). This internal driven capability is known as self-directed learning. Self-directed learning is described in terms of a person’s acceptance towards one’s responsibility for one’s own learning and a readiness to engage in developmental processes. Attitudes, abilities, and personality characteristics determine the readiness for self-directed learning (Fischer & King 2010:44).

Globally, healthcare services and professionals are bombarded with new discoveries and technological developments. The half-life of medical knowledge is seven to ten years. The shortened life span of relevant information highlights the necessity of healthcare professionals to keep abreast of new information, trends and developments in medical technology (Shen, Chen & Hu 2014:1).

## Problem statement

Cadorin *et al*. (2012:154) confirm the authors’ experience that there is scant evidence of the monitoring of self-directed learning processes and outcomes of healthcare professionals. The authors could not find any studies done on self-directed learning of nurse educators specifically, but identified and reviewed studies on the self-directed learning of preceptors. A strong internal locus of control as well as a high level of self-directed learning readiness are significant predictors of preceptor teaching competence (Chen *et al*. 2012:143).

Although there was a significant move towards student-centred learning internationally (Klunklin *et al*. 2010:177), Guglielmino and Toffler (2013:10) report that most educators still adhere to the behaviouristic teaching paradigm. It is our observation that didactic teaching is still the most dominant teaching mode in most nursing schools across the country and/or continent. Large class sizes, students’ expectations, and the need to cover a certain volume of content may contribute to the fact that most nurse educators still adhere to the traditional teaching method. Adherence to didactic instruction methods may also be due to the fact that educators imitate the way in which they were taught and because it is convenient; it does not take that much effort to use and adapt existing lecture and assessment material (Guglielmino & Toffler 2013:10). The aforementioned confirms that nurse educators do not follow current trends in higher education to be more student-centred. Furthermore, Fisher, King and Tague (2001:517) conclude that those who have a low self-directed learning approach experience high levels of anxiety when exposed to situations where they need to direct their own learning. Williamson (2007:67) concurs with Fisher and colleagues by stating that persons who are not strongly motivated to take the responsibility to improve on their knowledge and skills may lack self-confidence and fear failure. This may be a reason why nurse educators adhere to teaching methods that are convenient to them even though they have been shown to be the least effective.

These observations are no different for nurse educators. If nurse educators are not developed as self-directed learners they are not able to set the example and prepare students as independent learners. The cycle of following the same pattern as one’s predecessor is therefore continued. The implications are that once the nursing and midwifery students are professionals they will lack the knowledge and skills on how to keep abreast of trends in their profession. It is the responsibility of professional midwives and nurses to lead the way and to generate innovative ways of obtaining new knowledge and skills to the benefit of nursing and midwifery practice. The inability of the professional midwife and nurse to keep abreast with new developments in their profession will ultimately impact negatively on the quality of nursing and midwifery care.

Self-directed learning is of importance in the professional development of nurse educators as well as their professional conduct in the teaching and learning environment. As self-directed learners, educators will be able to support students more effectively. However, a challenge for today’s nurse educators is to develop into self-directed learners, to uphold and continue the lifelong learning approach and to guide students in becoming goal-directed and self-directed learners. The question therefore arises as to how the process of self-directed learning of nurse educators manifests in their role as educators?

## Aim of the study

The aim of the article is to describe the manifestations and implications of the lack of, or low levels of, self-directed learning of nurse educators.

### Definition of key concept

Knowles (1975:18 as cited by Yuan *et al*. 2012:427) defines *self-directed learning* as a process in which individuals take the initiative with or without the help of others to diagnose their learning needs, formulate learning goals, identify human and material resources for learning, choose and implement strategies and evaluate learning outcomes. Montin and Koivisto (2015:1) emphasise that self-directed learning is under control by the individual self. The definition by Knowles comprises actions that are associated with the domains of volition, motivation, self-management, self-monitoring, (Yuan *et al*. 2012:427). Shen *et al*. (2014:2) added communication as a construct of a lifelong learning process for nurses. Synonyms for self-directed learning are lifelong learning, active or independent learning, and student-centred education (Cadorin *et al*. 2012:153; Kocaman, Dicle & Ugur 2009:286). For the purpose of this article, self-directed learning will comprise four constructs, namely learning motivation, planning and implementing, self-monitoring and interpersonal communication. These constructs are explained in the discussion section of this article.

### Contribution to field

The value of this study is that nurses, midwives and nurse educators may recognise the paramount importance of self-directed learning for healthcare professionals, recognise the typical manifestations of low levels of self-directed learning and realise the implications of such behaviour. It further provides some food for thought with regard to one’s own readiness to engage in self-directed learning.

### Literature review

Educational trends have moved from passive learning to active learning, from superficial to deep learning, and from teacher-dependent learning to self-directed learning. An individual’s inclination towards self-directed learning may vary on this continuum of learning according to his or her interest, curiosity, self-concept as an effective student, future orientation, openness to learning opportunities, and acceptance of responsibility for meeting his or her own learning needs (Klunklin *et al*. 2010:177; Shen *et al*. 2014:1). Someone who is not interested in the topic or field will exhibit low self-directed learning in that specific field. However, a person who is interested in the topic will typically want to know more and therefore read more than the minimum prescribed works and will explore the topic in greater depth. Such people become autonomous in creating knowledge, perform academically better, are intrinsically motivated, gain confidence, have higher levels of personal and job satisfaction, are more resilient and open to change (Ali & Sebai 2010:189; Guglielmino & Toffler 2013:7-10; Montin & Koivisto 2015:1; O’Shea 2003; Shen *et al*. 2014:1; Yuan *et al*. 2012:427; Zhang *et al*. 2012:570).

O’Shea (2003:67) found that although educators are generally supportive of self-directed learning they doubted their ability to implement the processes and skills to attain these in a curriculum. Abraham *et al*. (2011:395) support O’Shea’s finding by concluding that, whilst nurse educators may have a strong desire to learn and be able to identify their learning needs they may face challenges regarding identifying the most relevant sources and how to manage their time and strategies. One of the tenets of constructivism that underpins self-directed learning is that learning is a social process. Williamson (2007:68) supports O’Shea (2003:67) on the viewpoint that self-directed learning is a collaborative process between the learning facilitator and the student. Teaching and learning strategies that support the development of identifying learning needs, managing resources and time, and monitoring own learning are: student-centred approaches, small group learning (Yuan *et al*. 2012:428), problem-based learning (Ali & Sebai 2010:188), computer-mediated learning, learning contract, independent learning projects (Guglielmino & Toffler 2013:7), distant education and teleconferencing (O’Shea 2003:64). These strategies are more appropriate for students that are registered in formal educational programmes than when one is mainly self-responsible for one’s daily activities such as nurse educators. Williamson (2007:68) emphasises the important role and responsibility that the nurse educator has in guiding a student to develop a lifelong learning approach.

## Research method and design

### Design

An instrumental case study design is used to gain insight into (Crowe *et al*. 2011) the manifestations and implications of self-directed learning of nurse educators. Baxter and Jack (2008:544) define a case as a ‘phenomenon of some sort occurring in a bounded context’ which makes case studies ideal to explore or describe a phenomenon in context. In this study the case is built around the feedback by programme participants and the educators’ responses as detailed in the case provided below (see Box 1).

**BOX 1:** Feedback by programme participants and the educators’ responses.

**Case** The University of Complex offers various nursing programmes. One of these programmes focused on student support strategies. Students who follow this programme are mainly professional nurses and nurse educators. The programme has been registered as a short learning programme with the university and all relevant regulatory bodies. Mrs Zulu and Miss Smith are the nurse educators responsible for presenting the short course. Mrs Zulu obtained her master’s degree in 2000 and Miss Smith completed her qualification in nursing education in 1991. She has no plans to continue with postgraduate studies as she will be retiring in 2018.
Dr Naidoo, the new coordinator of tuition in the nursing department insisted on programme evaluation by the attendees. She now routinely discusses programme evaluations with the presenters. After the 2014 programme she specifically discussed the following comments with them:
Comment 1: ‘We really like the lecturers but they have stagnated when it comes to their knowledge. We do not want to hear what they were doing in the olden days. We want the latest information, surely they should move on.’
Comment 2: ‘I am shocked, she did not even know much about the peer learning way of support.’
Comment 3: ‘I want to know how I can plan my own professional development to make sure that it is focused on my specific job requirements.’
Comment 4: ‘How will I know what or where to develop myself? I want to make sure that I know how to support my students who are Generation Ys. I have to understand what they want from us. I want to be in touch with my students to support them to perform well.’
In response to the feedback Mrs Zulu and Miss Smith mentioned that they developed all the teaching material together in 2008 and that this was the first time that there are any complaints about what and how they present the programme. Both lecturers strongly oppose the practice of evaluating programmes as they see it as an opportunity to complain anonymously. Miss Smith responded by stating that peer learning is a fashion word for student gatherings in the name of learning. Mrs Zulu appeared irritable but admitted that she needs to explore more innovative ways of supporting students. They could not understand the need to plan one’s own professional development because they see it as being the employers’ responsibility to develop staff to ensure that the employers are up to date with new trends and technology. Both presenters stated that Dr Naidoo must arrange for them to attend a capacity building workshop.

The role of the case is supportive and of secondary interest because it serves as a scenario in the exploration of manifestations and implications of self-directedness or the lack of self-directedness of nurse educators. The propositions of the case are that:

- The impetus to learn must come from the individual (Cadorin *et al*. 2012:154; Chen *et al*. 2012:143; Yuan *et al*. 2012:429).
- A self-directed learner self-identifies sources and manages his or her learning (Cheng *et al*. 2010; Shen *et al*. 2014:4; Yuan *et al*. 2012:429).
- The individual self-monitors progress (Cheng *et al*. 2010; Shen *et al*. 2014:4; Yuan *et al*. 2012:429).
- Knowledge and understanding of one’s own and students’ self-directed learning abilities are critical for nurse educators (Yuan *et al*. 2012).
- Supporting students to adapt to and predict change in today’s healthcare environment requires the skills for self-directed learning (O’Shea 2003).

### Data collection method

Based on the critical incident technique, data from various incidents over the past years were used to construct a fictitious case study. An incident is regarded as critical if it makes a significant contribution to the general aim of the activity, or in this case a phenomenon. Qualitative health researchers regard the critical incident as a well-proven approach that offers a practical step-by-step way to collecting and analysing information relating to human actions and its significance to the people involved (Hudges 2007).

Various critical incidents during the authors’ experiences as educators provided authentic foci which promoted an understanding of the phenomenon of self-directed learning. Both authors involved in constructing the case had real-life experiences similar to the scenario which added richness and depth to the fictitious case presented below Specific information from critical incidents were purposively selected to construct the fictitious case. In constructing the fictitious case the authors used reflection as a way to explore, explain, consider, justify and learn from the specific incidents that occurred in their practice. The feedback comments, as well as the responses of the educators as depicted in the case, are real.

### Data analysis

The authors deductively analysed the case by using the literature on self-directed learning readiness as departure point. The three propositions (learning motivation, planning and implementing, self-monitoring) have been confirmed in exploratory and confirmatory factor analysis of various self-directed learning readiness questionnaires (Cheng *et al*. 2010:1152; Fisher & King 2010:46-47; Shen *et al*. 2014:4; Williams & Brown 2013:430; Yuan *et al*. 2012:429). The authors’ interpretation of the programme participants’ feedback and comments was that the programme presenters were not keeping abreast with the developments within the field of student support. Subsequently, the presenters exhibited low self-directed learning which was confirmed by some remarks in the discussion on the participants’ comments. The analysis process followed the steps of deductive reasoning as described in Bruce, Klopper and Mellish (2011:162), namely to start with the theory of self-directed learning as defined by Knowles (1975), state a hypothesis and to confirm the hypothesis through observation and other sources of evidence. With the deductive approach used in this article, instead of confirming an hypothesis, the propositions were the elements that were examined within the scope of the study (Yin 2014:30). Furthermore, the analysis was an iterative process (Baxter & Jack 2008:548) because the authors interpreted the statements in the case and categorised it as a self-directed learning domain. The statements or questions in the validated questionnaires were then read to check if the interpretation fitted the tested domain items. Through discussion the authors reached consensus on the item that best suited the case statement and only then finalised the categorisation of the comments.

### Context of the study

The context is of educators at a nursing department within a higher education institution in Sub-Saharan Africa because the authors experienced the scenario depicted in the case in a number of countries in the region and in different nursing departments.

## Ethical considerations

Researchers take responsibility for the integrity of the interpretation and presentation of the results. Although the case in this study is fictitious the comments and responses are based on real-life experiences of the authors. Pseudonyms are used to protect people and institutions. As the case in instrumental case studies is of lesser importance than in other case study designs, the authors are of the opinion that the constructed case serves the purpose of the study design. The researchers maintained scientific integrity in all phases of the research to prevent or minimise bias. The authors hold an intellectual position that this study views knowledge as being intellectual as well as action. Therefore, the relationship of knowledge and action when creating the case is within the ethical realms of social research (Babbie & Mouton 2001:537).

## Trustworthiness

Trustworthiness was ensured regarding neutrality of the findings and conclusions made in the study. Credibility was achieved through prolonged engagement over a couple of years as educators in the constructed case. Through persistent observation the authors reached consensus on the interpretations that best suited the case statement and only then finalised the categorisation of the comments. A peer who has experienced similar critical incidents but who is outside of the study reviewed the insights, perceptions and analyses gained from the case study. As the case study was constructed from real-life incidents it could be transferable to other contexts. Purposive sampling of specific information from the critical incidents was used to maximise the range of information related to self-directed learning. Should the study be repeated, the evidence provided in that study would be similar in that the data analysis was based on valid and reliable self-directed learning readiness questionnaires. Confirmability was assured in the audit trail that remained when determining if the conclusions, interpretations and recommendations were indeed based on the existing self-directed learning readiness questionnaires.

## Results and discussion

**FIGURE 1:**
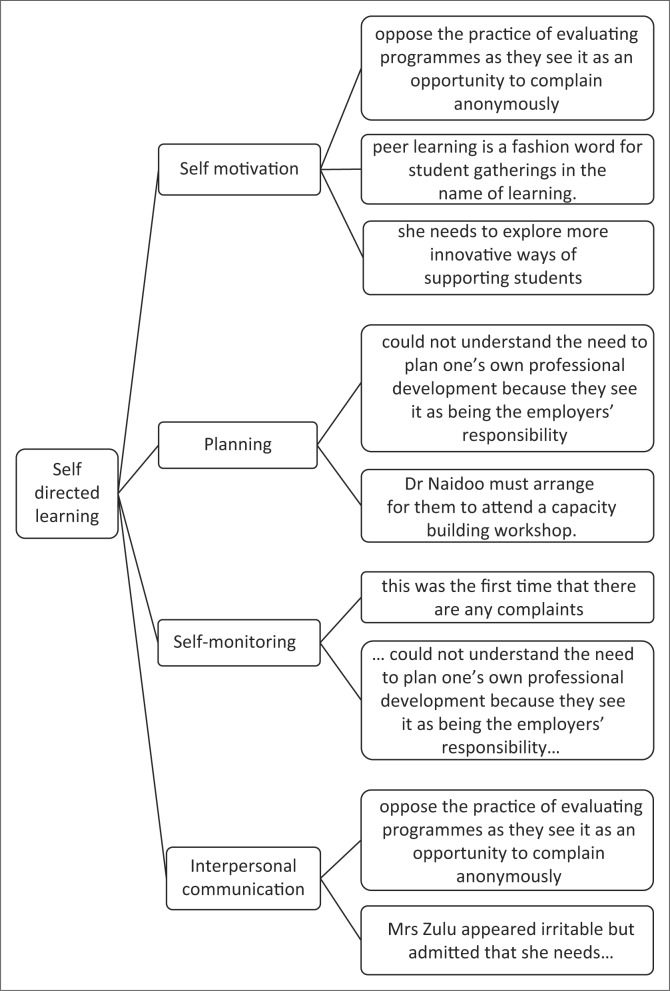
Analysis of the responses of the participants.

The findings of the analysis are depicted as real responses. A total of nine responses illustrated in the case study were analysed. Although the responses were categorised according to the four constructs of self-directed learning, they are not necessarily exclusive to one construct but show an interrelationship within the four constructs (Figure 1).

The aim of the article is to describe the manifestations and implications of the gap in self-directed learning of nurse educators based on the four constructs of the self-directed learning readiness questionnaire. Evidence from examining the real responses in the case through an iterative process of deductive reasoning the data were categorised into these four constructs. By examining the real responses in the case using an iterative process of deductive reasoning, categorising of the data into these four constructs was enabled. Consensus was reached amongst the authors on the item that best suited the case statement, which then confirmed and finalised the categorisation of the comments. Further to the discussion of the real comments, ideal comments that are congruent with a self-directed learner or individual are provided.

### Learning motivation

Learning motivation refers to the inner drive of the individual as well as external factors that motivate learning and stimulate a sense of responsibility for one’s own learning. The educators in the fictitious case study opposed the practice of evaluating programmes as they saw it as an opportunity for students to complain anonymously. They were not able to identify their shortcomings in what they needed to learn. A self-directed individual would have acknowledged the value of such a comment as it could be regarded as a motivation to improve in their learning. Facing some difficulty, such as having to respond to an anonymous evaluation, should not hinder the motivation to learn. Character traits of self-directed educators include having an internal locus of control, hence achieving desired goals through their own efforts (Chen *et al*. 2012:142). In the case provided the educators were not able to reflect on the comments and apply them consciously to identify their learning needs to decide on their own growth and development.

A response that shows readiness to self-directed learning would have included the acknowledgement that the practice of evaluating programmes is an opportunity to serve as pointers and drivers to persist, despite some difficulties, and to find solutions to challenges and concerns. It creates prospects of assessing what learning shortcomings exist and the effectiveness of one’s learning processes.

### Planning and implementing

The ability to plan and implement learning methods, learning strategies and skills is necessary to effectively implement the process of self-directed learning (Shen *et al*. 2014:6). The educators in the scenario could not understand the need to plan their professional development because they saw it as being the employers’ responsibility. They felt that the programme coordinator should have arranged for them to attend a capacity building workshop. A self-directed learner can pro-actively establish his or her learning goals and set priorities that will be followed and controlled based on own needs (Fisher & King 2010:47).

Persons with self-directed learning readiness would have responded by acknowledging that they should set priorities to reach self-identified goals to improve on the shortcomings identified in their learning. They should then determine the specific type of capacity building session, time and resources needed to reach the learning goals. Cheng *et al*. (2010:1153) describe this construct as ‘self-management’ in diagnosing learning needs, formulating learning goals, identifying human and material resources for learning and choosing and implementing appropriate learning strategies.

### Self-monitoring

Self-monitoring entails learning evaluation by assessing one’s learning outcomes (Cheng *et al*. 2010:1153). The responses of the educators in the scenario clearly indicated that they regard the evaluation of their performance and strengths and weaknesses as unjustified complaints. They could also not understand the need to plan one’s own professional development because they saw it as being the employers’ responsibility. They were not able to identify the need to connect knowledge and personal experience with their strengths and weaknesses, which in turn serves as evaluation of learning outcomes.

The response that would have been expected by a self-directed learner should have included an acknowledgement that the evaluation serves as an additional way of identifying their strengths and weaknesses in monitoring their learning progress. The experience of going through an evaluation should, or could, also serve as a personal developmental experience that could be linked to an understanding of their level of knowledge (Shen *et al*. 2014:4).

### Interpersonal communication

Communication is a key to successful education. Imparting knowledge entails two-way communication. The interaction with students helps educators to plan for own further learning to ensure effective teaching through both the spoken word and through role modelling and interaction. By opposing the practice of evaluating programmes as they perceived it as an opportunity for students to complain anonymously, the value of communication is disregarded. By interacting with others, one is able to identify shortcomings and plan for one’s further learning. Through the process of two-way communication the educators in the case provided could also have obtained a better understanding of the specific needs of the students.

The appropriate response by self-directed learners would have been to acknowledge the willingness and openness of the students to openly communicate their concerns related to the programme and to regard the feedback as constructive and guiding criticism.

### Limitations of the study

Most articles reviewed are about the self-directed learning readiness of students with only a few focusing on the self-directed learning readiness of practicing healthcare professionals. A verbatim transcription of a similar real-life experience as the fictitious case may have added to the authenticity of the case. However, the fictitious case was built on real-life experiences of the authors. The focus was therefore not on one real-life incident but rather extracted information from various critical incidents.

### Recommendations

Recommendations on how educators could promote self-directed learning readiness during the training and practice of nurses and midwives are listed below.

Nurse and midwifery educators should:

- Foster an appreciative approach to self-directed learning by creating innovative ways of celebrating self-directedness. Celebrate progress and recognise success.
- Build a climate supportive of self-directed learning through the promotion of lifelong learning. Respect individual strengths and needs and value, acknowledge and accommodate passion for, and excitement in, learning.
- Promote individual awareness of the self as a self-directed learner by acknowledging individual differences in the learning process as expressed in learning styles.
- Encourage continuous assessment of self-directed learning readiness and create opportunities to celebrate achievements. Provide a safe environment within which weaknesses could be addressed.
- Provide a safe environment within which self-directedness could be determined using practical and applied learning opportunities. Role play or group interviews are some examples.
- Build in transition structures in the process of developing self-directed learning processes. Examples could be to offer a variety of ways to demonstrate successful performance or to use activities that will give individuals an opportunity to take on an increasing number of responsibilities as educators.
- Use innovative and generative learning approaches to stimulate creativity amongst students.
- Introduce a variety of teaching or educational approaches, such as problem-based learning, field-based learning or project-based learning.
- Provide support systems through mentoring programmes, peer support strategies and needs assessment techniques.
- Encourage and foster open communication between educators and students to ensure honesty and constructive feedback in a non-threatening environment.
- Encourage metacognition, reflective practices, visualisation and thoughtful evaluation.
- Use assessment strategies that build self-directed learning skills and abilities.

## Conclusion

There is a growing trend of preparing self-directed learners in the field of nursing. However, if the educators themselves are not self-directed in their own learning they are not able to support students in becoming self-directed learners (Cheng *et al*. 2010:1153). A fundamental feature in the success of lifelong learning is the ability to engage in self-directed learning. Although various reliable and valid instruments exist to determine self-directed learning readiness, these instruments are not used on a regular basis, especially amongst nurse educators.

Self-directed learning is multi-dimensional (Shen *et al*. 2014:4). It requires openness to learning opportunities, good self-concept, initiative and independence in learning and a love for learning. Conscientiousness, an informed acceptance of a responsibility for one’s learning and creativity are key to a person’s future orientation towards learning. In the context of higher education and the nursing profession, self-directed learning is of boundless importance to the professional development of its educators and students. Current and emerging trends in the health care environment, medical technology and scientific world urge all role players to be more flexible, open to change, remain current in their knowledge and skills and maintain a high level of professional confidence.
